# Rapid CRISPR–Cas9 target-strand nicking can provide phage resistance by reducing DNA abundance

**DOI:** 10.1093/nar/gkaf900

**Published:** 2025-09-23

**Authors:** Giang T Nguyen, Akshara Raju, Michael A Schelling, Dipali G Sashital

**Affiliations:** Roy J. Carver Department of Biochemistry, Biophysics and Molecular Biology, Iowa State University, Ames, IA 50011, United States; Roy J. Carver Department of Biochemistry, Biophysics and Molecular Biology, Iowa State University, Ames, IA 50011, United States; Roy J. Carver Department of Biochemistry, Biophysics and Molecular Biology, Iowa State University, Ames, IA 50011, United States; Roy J. Carver Department of Biochemistry, Biophysics and Molecular Biology, Iowa State University, Ames, IA 50011, United States

## Abstract

Cas9 is an RNA-guided immune endonuclease that provides bacterial defense against bacteriophages. Cas9 relies on divalent metal ions for cleavage catalysis by two domains, HNH and RuvC, and to facilitate conformational changes that are required for cleavage activation. While Cas9 typically produces double-strand breaks (DSBs) in DNA targets, we observed that reduced, physiologically relevant Mg^2+^ concentrations can result in a slow rate of non-target strand cleavage by RuvC. This raised the question of whether rapid target-strand nicking by the Cas9 HNH domain is sufficient to provide protection against phage. To address this, we tested phage protection by Cas9 nickases, in which only the HNH or RuvC domain is catalytically active. We find that nicking by HNH, but not RuvC, can be sufficient to provide immunity. Target-strand nicking prevents phage DNA accumulation and can reduce the susceptibility of Cas9 to viral escape. Cleavage by RuvC is strongly impaired in the presence of other biomolecules that can compete for binding of free Mg^2+^, preventing formation of a DSB. Overall, our results suggest that HNH cleavage may occur more rapidly than RuvC cleavage under physiological conditions, resulting in an initial target-strand nick that may be sufficient to provide CRISPR-mediated immunity.

## Graphical Abstract

**Graphical Abstract:**
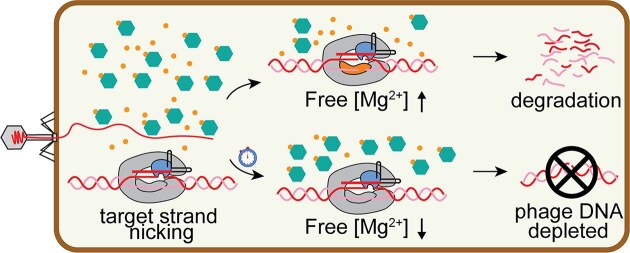

## Introduction

CRISPR–Cas (clustered regularly interspaced short palindromic repeats-CRISPR associated) systems provide sequence-specific protection to bacteria and archaea against viral infection [1–4]. CRISPR arrays encode CRISPR RNAs (crRNAs) containing “spacer” sequences with complementarity to viral genomes [5–7]. Cas effectors use these crRNAs as guides to find and destroy complementary nucleic acids upon infection [2, 3]. New spacers can be acquired into the CRISPR array allowing for adaptation against specific viruses [1].

Cas effectors vary widely in their composition and mechanisms of action [8]. Cas9 and Cas12a effectors target double-stranded DNA (dsDNA) and are composed of a single Cas endonuclease [9–11]. Targeting dsDNA requires a protospacer adjacent motif (PAM) that is located next to the spacer-complementary target region [9–13]. Cas endonucleases initially recognize PAM sequences and directionally unwind DNA away from the PAM, allowing for formation of an RNA–DNA hybrid between the crRNA spacer and the target strand of the DNA [14, 15]. Formation of this R-loop enables cleavage by Cas endonuclease domains [16, 17]. Cas9 contains two metal-dependent nuclease domains, HNH and RuvC, each of which cleaves a DNA strand to generate a double-stranded break (DSB) [9, 10, 16]. The HNH domain cleaves the crRNA-complementary “target” strand using a one-metal-ion-dependent mechanism, while the RuvC domain cleaves the opposite “non-target” strand via a two-metal-ion-dependent mechanism [9, 18]. Cleavage by the two Cas9 domains can occur simultaneously [16], but has also been observed to occur sequentially with the HNH domain cleaving prior to RuvC, potentially due to a two-step formation of the R-loop [18]. Active site mutations in either of these domains convert Cas9 into a nickase, which cleaves only a single strand in the DNA target [9, 10]. Cas12a only contains a single RuvC nuclease domain, which is used to cleave the non-target and target strands in a sequential manner [19, 20].

In addition to serving as catalytic cofactors, metal ions are required for additional steps in the overall mechanisms of Cas9 and Cas12a. Mg^2+^ ions affect binding of both the guide RNA and the target DNA to Cas endonucleases [21–25]. The Cas9 HNH domain requires divalent cations to transition from an inactive to an active conformation following target binding [26, 27]. RuvC cleavage is modulated allosterically by these conformational changes of the HNH domain, but not by HNH nuclease function [16]. Cas12a conformational changes also depend on metal ions. Mg^2+^-mediated local DNA unwinding in the PAM-distal region is required prior to the second, target-strand cleavage event by Cas12a [28].

Importantly, the physiological concentration of free Mg^2+^ is 1–2 mM in bacteria [29, 30]. Previous studies have suggested that Cas endonucleases have defects in second strand cleavage at physiologically relevant Mg^2+^ concentration and especially upon introduction of PAM-distal mutations in the target DNA [24, 28, 31–35], which can occur in native settings when viral targets mutate under pressure of CRISPR–Cas immunity [13, 36–41]. However, single point mutations in the PAM-distal region are rarely sufficient to allow complete escape from Cas9 or Cas12a. Cas9 has also been observed to have reduced rates of second-strand cleavage when using perfectly matched plasmid targets [35, 42, 43], a defect that is exacerbated at lower Mg^2+^ concentrations [44]. Together, these observations raise the question of whether and how slow rates of second-strand cleavage by Cas endonucleases affect antiviral protection in cells.

To address this question, we compared the efficacy of wild-type (WT) Cas9 and Cas9 nickases in providing anti-bacteriophage protection in *Escherichia coli*. We accumulated a comprehensive dataset using >60 guide RNAs targeting either strand of the λ_vir_ genome in coding and non-coding regions with varying essentiality. We find that nicking by the HNH domain, but not the RuvC domain, can provide protection at a similar level to WT Cas9 for a subset of targets, although the degree of protection by both WT Cas9 and the HNH nickase varies depending on target location. Phage proliferation is inhibited by both WT Cas9 and the HNH nickase, while the RuvC nickase has little effect on the amount of phage DNA that accumulates over time. Using *in vitro* biochemistry, we show that the RuvC nickase, but not the HNH nickase, can be readily turned over by RNA polymerase. In addition, cleavage by the RuvC nickase is strongly impaired at low Mg^2+^ concentration, especially in the presence of biomolecules that can compete for metal ion binding. Overall, our results suggest that the low Mg^2+^ concentrations present in cells may result in rapid nicking of target DNA by the Cas9 HNH domain and delayed formation of DSBs due to impairment of the RuvC domain.

## Materials and methods

### Expression plasmid construction

Primers used for Cas9 mutagenesis and single-guide RNA (sgRNA) plasmid construction are listed in Supplementary Tables S1–S3. SpCas9 expression plasmids and sgRNA expression plasmids were constructed using pACYCDuet-1 and pUC19, respectively. Both Cas9 and sgRNA were inserted downstream of a pBAD promoter as previously described [36]. Mutations were introduced into the Cas9 construct by round-the-horn polymerase chain reaction (PCR) and ligation to create the HNH (D10A) or RuvC (H840A) nickases. A second mutation was introduced to produce the dCas9 expression construct (D10A, H840A).

For Cas9 purification, the expression vector pMJ806 was a gift from Jennifer Doudna (Addgene plasmid #39312; http://n2t.net/addgene:39312; RRID:Addgene_39312) [9]. The D10A and H840A substitutions were introduced via round-the-horn for expression of the HNH and RuvC nickase, respectively.

### Phage propagation and stock preparation

A lytic mutant of phage λ (λ_vir_) [45] and Mu phage [46] were obtained from the Félix d’Hérelle Reference Center for bacterial viruses of the Université Laval. To prepare λ_vir_, 2 mL of *E. coli* BW25113 culture was grown from a single colony in LB media supplemented with 10 mM MgSO_4_ to OD_600_ of 0.5 at 37°C with shaking. A total of 300 μL of the culture was infected with 20 μL of diluted phage stock and mixed with 3 mL of soft LB agar (LB media supplemented with 0.7% agar and 10 mM MgSO_4_) and poured onto LB plates. The plates were grown overnight at 37°C. Individual plaques with soft agar were picked into 50 μL of deionized H_2_O and incubated at 48°C for 20 min to allow the agar to dissolve. The picked plaques were then added to a 2 mL culture of *E. coli* BW25113 cells grown in LB supplemented with 10 mM MgSO_4_ at an OD_600_ of 0.5. The cultures were grown at 37°C for 2–3 h until lysis was observed. A few drops of chloroform were then added and the samples were centrifuged at 5000 rpm for 5 min. The supernatant was separated and stored as phage stocks at 4°C, which was used to prepare higher titers of phage by adding 10 μL of phage to 2 mL of *E. coli* BW25113 at OD_600_ of 0.5 grown in LB + 10 mM MgSO_4_. Following clearing of the culture, the phage was prepared using chloroform as described earlier.

Mu phage was prepared similarly to above with the following modifications. *Escherichia coli* BW25113 grown in 2 mL LB media supplemented with 10 mM MgSO_4_ were grown at 37°C with shaking for 30 min. Individual phage plaques or 10 μL of Mu phage stock were added to the cultures, which were grown for another 90 min. The temperature of the shaker incubator was then changed to 44°C for 20 min then returned to 37°C. The cultures were incubated for another 2 h, and the lysate was harvested using chloroform and stored as described earlier.

### Phage spot assays

All targets for phage assays are listed in Supplementary Tables S1 and S2. Cas9 and sgRNA plasmids were transformed into *E. coli* BW25113 cells and plated onto LB media (1.5% agar, 100 μg/mL ampicillin, 25 μg/mL chloramphenicol). Colonies were picked and inoculated into 2 mL LB media (100 μg/mL ampicillin, 25 μg/mL chloramphenicol, and 10 mM MgSO_4_) and allowed to grow overnight at 37°C in a shaker incubator (Thermo Scientific™ MaxQ™ 4000 Benchtop Orbital Shakers). These overnight cultures were then mixed with fresh 2 mL LB media in 1:100 ratio (100 μg/mL ampicillin, 25 μg/mL chloramphenicol, 20 mM arabinose, and 10 mM MgSO4) and the cultures were grown at 37°C. When cells reached an OD_600_ of 0.5, 300 μL were mixed with soft LB agar (0.7% agar, 100 μg/mL ampicillin, 25 μg/mL chloramphenicol, 20 mM arabinose to induce protein expression, and 10 mM MgSO_4_) and overlaid on LB plates with respective antibiotics. The LB agar was then allowed to solidify for 15 min at room temperature before spotting the phage. A 10-fold dilution series of phage (titer of 1.4 × 109 PFU/mL) was prepared and 2 μL of each dilution was spotted on the bacterial lawn. Plates were incubated at 37°C overnight for the plaques to form. Plaques were imaged the next day.

### Liquid culture phage assays and growth curves

The phage challenge assays were conducted using *E. coli* strain BW25113, which is a derivative of K12 [47]. Cas9 and crRNA expression plasmids were introduced into *E. coli* by heat shock. The transformed cells were plated on LB agar plates containing ampicillin (100 μg/mL) and chloramphenicol (25 μg/mL) for selection. A single colony was cultured overnight in LB media with the same antibiotics as above. Fresh 200 μL LB cultures supplemented with 100 μg/mL ampicillin, 25 μg/mL chloramphenicol, 20 mM arabinose, and 10 mM MgSO_4_ were inoculated with the overnight cultures in a 1:100 ratio. Lambda phage was added at MOI 1 at OD_600_ of 0.6. The cultures were grown in a TECAN Infinite M Nano+ 96-well plate reader at 280 rpm and 37°C, and OD_600_ measurements were taken every 10 min.

### Cell viability assays

Cell viability assays were performed at least three times for each guide shown in Supplementary Fig. S2. For each experiment, six separate cultures were inoculated as described in the previous section. Three cultures were used for assessing cell viability and the remaining cultures were used to assess growth curves. All cultures were recorded in the same 96-well plate. At OD_600_ of 0.5, 200 μL of culture was removed from one of the samples for each strain. Phage was added to the rest of the cultures at an MOI of 1. Two additional cultures were harvested at 1 and 4 h post-infection for each strain. Immediately following removal of the cultures at each time point, cells were pelleted and washed twice using 200 μL fresh LB media to remove phage in the media. The washed cultures were then resuspended in 200 μL fresh LB, serially diluted tenfold, and plated on LB media supplemented with antibiotics. The plates were incubated at 37°C overnight. The colonies were imaged the next day.

### WT Cas9 and Cas9 nickase protein expression and purification

The expression of Cas9 proteins was carried out in *E. coli* BL21(DE3) cells. To initiate the expression, an overnight culture of the cells carrying the respective expression plasmid was added into fresh LB media (at a 1:100 ratio) supplemented with 50 μg/mL kanamycin. When the culture reached an OD_600_ of 0.5–0.6, protein expression was induced by the addition of 0.5 mM IPTG. The induced culture was then incubated at 18°C for ∼16 h with continuous shaking. Cells were centrifuged at 6000 × *g* for 15 min at 4°C.

Harvested cells were resuspended in lysis buffer containing 20 mM Tris–HCl (pH 8.0), 500 mM NaCl, 5 mM imidazole, and 5% glycerol. One millimolar phenylmethylsulfonyl fluoride (PMSF) was added before sonication and the lysate was centrifuged to remove insoluble material. The clarified supernatant was transferred to a HisPur Ni-NTA resin column (Thermo Fisher Scientific) pre-equilibrated with lysis buffer. The column was washed with 50 column volumes of lysis buffer, followed by a second wash with 50 column volumes of wash buffer [20 mM Tris–HCl (pH 8.0), 500 mM NaCl, 15 mM imidazole, and 5% glycerol]. An elution buffer composed of 20 mM Tris–HCl (pH 8.0), 500 mM NaCl, 250 mM imidazole, and 5% glycerol was applied. The eluted protein fractions were collected and analyzed by sodium dodecyl sulfate–polyacrylamide gel electrophoresis (SDS–PAGE). Fractions containing MBP-Cas9 were combined and cleaved with TEV protease in a 1:100 (w/w) ratio to remove the MBP tag at 4°C while dialyzing against dialysis buffer [10 mM HEPES-KOH (pH 7.5), 200 mM KCl, 1 mM DTT, and 5% glycerol]. After overnight dialysis, the protein was diluted with a buffer containing 20 mM HEPES-KOH (pH 7.5) and 5% glycerol to a final concentration of 100 mM KCl. The protein was loaded on a HiTrap Heparin HP (Cytiva) column, which was pre-equilibrated with Buffer A [20 mM HEPES-KOH (pH 7.5), 100 mM KCl, and 5% glycerol]. Twenty percent of buffer B [20 mM HEPES-KOH (pH 7.5), 1 M KCl, and 5% glycerol] was applied to wash the column. A gradient from 20% to 100% of buffer B was applied over a total volume of 60 mL to elute. Protein fractions were collected and analyzed using SDS–PAGE. Fractions containing the protein of interest were pooled and concentrated to a final volume of 1 mL. The concentrated protein was diluted to 15 mL with storage buffer [20 mM HEPES-KOH (pH 7.5), 200 mM KCl, and 1 mM DTT]. This buffer exchange step was performed three times. Finally, the concentrated protein was aliquoted, flash-frozen in liquid nitrogen, and stored at −80°C for future use.

### T7 RNA polymerase expression and purification

T7 RNA polymerase (T7 RNAP) expression and purification were performed by the following protocol. pTT7-911Q (T7 RNAP expression plasmid) was transformed into BL21(DE3) and plated on LB containing 50 μg/mL ampicillin. A single colony was picked and grown overnight in 25 mL LB medium supplemented with 50 μg/mL ampicillin. To initiate the expression, the overnight culture was added into fresh LB media (at a 1:100 ratio) supplemented with 50 μg/mL ampicillin and grown at 37°C to OD_600_ of 0.5–0.6. Protein expression was induced by adding 0.5 mM IPTG and then continued to grow at 37°C for 3 h. The cell pellets were harvested at 6000 x *g* for 15 min at 4°C.

The cell pellets were resuspended in lysis buffer containing 50 mM Tris–HCl (pH 8.0), 100 mM NaCl, 5 mM β-Mercaptoethanol, 1 mM imidazole, and 5% glycerol. 0.1 mM PMSF was added into the resuspended cells right before the sonication step and the cell lysate was then centrifuged at 20 000 × *g* for 30 min. The lysate was incubated with 2 mL HisPur resin (pre-equilibrated with lysis buffer) with gentle rocking for 1 h at 4°C. The resin was pelleted at 1000 x *g* for 2 min, then the lysate was carefully poured off. The resin was washed four times with lysis buffer and then four times with wash buffer (50 mM Tris, pH 8, 100 mM NaCl, 5 mM β-Mercaptoethanol, 10 mM imidazole, and 5% glycerol) using batch method. The protein was eluted using elution buffer (50 mM Tris, pH 8, 100 mM NaCl, 5 mM β-Mercaptoethanol, 200 mM imidazole, and 5% glycerol). After analyzing the collected fractions using SDS–PAGE gel, the fractions containing the protein of interest were pooled together and concentrated to a final volume of 1 mL. The concentrated protein was run on a Superdex 200 column (Cytiva) using SEC buffer (50 mM Tris, pH 8, 100 mM NaCl, 5 mM β-Mercaptoethanol, and 5% glycerol). Protein fractions were analyzed and dialyzed overnight using dialysis buffer (20 mM sodium phosphate, pH 7.7, 100 mM NaCl, 50% glycerol, and 1 mM DTT). Finally, the protein was aliquoted and stored at −20°C.

### crRNA and tracrRNA preparation

crRNA and tracrRNA were *in vitro* transcribed by the following protocol. For the crRNA, short oligonucleotides (Integrated DNA Technologies, Supplementary Table S4) consisting of a T7 promoter region, and a template sequence were initially pre-annealed to a complementary T7 promoter sequence by heating at 90°C for 2 min, followed by incubation at room temperature for 10 min. The tracrRNA template was cloned into the pUC19 plasmid, incorporating an EcoRI restriction site at the end of the template sequence (Supplementary Table S4). The tracrRNA plasmid was initially linearized using EcoRI and subsequently used as a template for *in vitro* transcription without the pre-annealing step. Transcription reactions (500 μL) were performed in transcription buffer containing 40 mM Tris (pH 8.0), 38 mM MgCl_2_, 1 mM Spermidine (pH 8.0), 0.01% Triton X-100, 5 mM ATP, 5 mM CTP, 5 mM GTP, 5 mM UTP, and 5 mM DTT. The reaction mixture contained 0.5 μM pre-annealed DNA or 35 μg plasmid DNA and 1 μM T7 RNAP. The transcription reaction was performed at 37°C for 4 h. Following the reaction, 2X RNA dye (New England Biolabs) was added, the mixture was heated at 95°C for 5 min, and then immediately cooled on ice. The reaction mixture was separated on a 10% polyacrylamide gel containing 1× TBE and 8 M urea. The crRNA band was visualized under UV light and excised from the gel. The gel slice was crushed and soaked overnight in 1 mL of nuclease-free water at 4°C with gentle agitation. After centrifuging the gel mixture for 5 min at 2000 × *g*, the crRNA solution was transferred to Costar Spin-X centrifuge tube filters (Sigma–Aldrich) and then centrifuged at the highest speed for 2 min to collect the crRNA solution in the collection chamber. crRNA was concentrated by ethanol precipitation, aliquoted, and stored at −20°C.

### *In vitro* cleavage assays

Cleavage assays were performed as previously described [36, 44]. Plasmid targets were constructed using restriction cloning with pUC19 using the oligonucleotides listed in Supplementary Table S4. The reaction buffer contained 20 mM HEPES (pH 7.5), 100 mM KCl, 1 mM or 10 mM MgCl_2_, 1 mM DTT, and 5% glycerol. The final concentration of SpCas9 was 50 nM. The RNP complex was formed by incubating Cas9, crRNA, and tracRNA at a ratio of 1:1.5:1.5 at 37°C for 10 min. To initiate the cleavage reaction, pre-warmed target plasmid at 37°C was added to a final concentration of 15 ng/μL and a final reaction volume of 100 μL. The reaction was continuously incubated at 37°C and 10 μL of aliquots were quenched at 7, 15, 30, 60, 300, 900, and 1800 s by 10 μL phenol-chloroform-isoamyl alcohol (25:24:1 v/v, Invitrogen). Following extraction of the aqueous layer, the cleavage products were separated on a 1% agarose, stain-free gel and visualized using SYBR Safe by post-staining the gel (Invitrogen). The gels were quantified using ImageJ [48], and first- and second-strand cleavage were determined as previously described [44]. The data were fit to the appropriate rate equation (Supplementary Fig. S1) using GraphPad Prism. The first-strand cleavage and HNH nickase cleavage fit better to a double-exponential rate equation, yielding two rate constants and amplitudes fit for the fast and slow phases. We determined *k*_avg_ using the following equation:


\begin{eqnarray*}
{{k}_{{\rm avg}}}{\mathrm{\ }} = {\mathrm{\ }}{{A}_{{\rm fast}}} \times {{k}_{{\rm fast}}} + {{A}_{{\rm slow}}} \times {{k}_{{\rm slow}}}.
\end{eqnarray*}

The second-strand cleavage and RuvC nickase cleavage fit better to a single-exponential rate equation, yielding a single rate constant, reported as *k*_obs_.

For Cas9 turnover assays, the reaction buffer contained 20 mM HEPES (pH 7.5), 100 mM KCl, 10 mM MgCl_2_, 1 mM DTT, 1 mM rNTP, and 5% glycerol. The Cas9 RNP was formed as described earlier. The concentration of Cas9 was 10 nM and the final concentration of target plasmid was 20 ng/μL (∼12 nM). One micromolar of T7 RNAP was added 1 min after initiating the reaction. Ten microliter aliquots were quenched at 2, 5, 10, 20, 30, 50, and 70 min and separated on a 1% agarose gel with post-staining using SYBR Safe. The intensity of individual DNA bands was quantified using ImageJ software [48]. To determine the fraction of cleaved DNA, the total cleaved DNA (including nicked and linearized DNA) was divided by the total DNA (nicked, linearized, and supercoiled DNA). The fraction cleaved was plotted against time, and the data was fitted to a double-exponential equation in GraphPad Prism. Each measurement was performed in triplicate.

For cleavage assays performed in the presence of ATP, the reaction buffer contained 20 mM HEPES (pH 7.5), 100 mM KCl, 1 mM MgCl_2_, 1 mM DTT, various concentrations of ATP (0.1, 0.2, 0.5, 1, 2, 5, 10 mM), and 5% glycerol. The final concentration of SpCas9 was 50 nM and the RNP was formed as described earlier.

### qPCR sample preparation

The transformed cells were grown in LB medium supplemented with ampicillin (100 μg/mL) and chloramphenicol (25 μg/mL) to an OD_600_ of 0.5–0.6 and infected with lambda phage at MOI 0.2. The cultures were incubated at 37°C for 10 min without shaking to allow phage to inject their genetic material into the host cells. The cultures were centrifuged at 5000 × *g* for 5 min. The supernatant was discarded, and the cell pellets were resuspended in fresh LB medium and pelleted again as earlier to remove excess phage. The cell pellets were resuspended in fresh LB. One milliliter of this culture was harvested immediately (T0 samples) and the remaining culture was incubated at 37°C with shaking. At times = 30, and 60 min post-infection, 1 mL samples of the culture were harvested. To stop replication, the collected samples were heated at 100°C for 10 min. The samples were diluted 10× with nuclease-free water and directly used as template for quantitative PCR (qPCR).

### Quantitative PCR

qPCR reactions (20 μL) contained 1X SYBR green mix (Thermo Fisher), 500 nM of phage-specific primers or genome-specific primers, and 2 μL of prepared samples described earlier. QuantStudio3 Real-Time PCR system (Thermo Fisher) was used to amplify the DNA templates according to the following program: one cycle of 50°C for 2 min, one cycle of 95°C for 10 min, and 40 cycles of 95°C for 15 s and 60°C for 60 s. PCR reactions were performed using primers amplifying gene L in the λ genome or the glyceraldehyde-3-phosphate dehydrogenase (*gap*) gene from the *E. coli* genome (Supplementary Table S5).

DNA abundance was determined by quantifying the fold change in phage genome copy at *T* = 30 or 60 compared to *T* = 0. Ct values for gene L of the λ_vir_ genome were compared to Ct values for the *gap* gene from the host *E. coli* genome to account for differences in sample input or changes in cell growth following phage infection. Fold change was calculated using the following formula, assuming 100% amplification efficiency (*E* = 2) for both targets:

Fold Difference = 2^ΔCt_λ^ / 2^ΔCt_host^

where


\begin{eqnarray*}
\Delta Ct\_\lambda {\mathrm{ }} = {\mathrm{ }}Ct\_{\mathrm{ }}{{\lambda }_{0\min }}--{\mathrm{ }}Ct\_{{\lambda }_{30\_{\rm or}\_60\min }},
\end{eqnarray*}

\begin{eqnarray*}
\Delta Ct\_host{\mathrm{ }} = {\mathrm{ }}Ct\_{\mathrm{ }}hos{{t}_{0{\rm min}}}--{\mathrm{ }}Ct\_{\mathrm{ }}hos{{t}_{30\_{\rm or}\_60{\rm min}}}.
\end{eqnarray*}

Relative DNA abundance was calculated by dividing the fold difference in each experimental sample by the fold difference observed in a control sample expressing a non-targeting sgRNA. Three biological replicates were performed for each sample. Ct values are provided in Supplementary Table S6.

## Results

### Cleavage by the Cas9 RuvC domain is strongly impaired at low Mg^2+^ concentration

**Figure 1.**
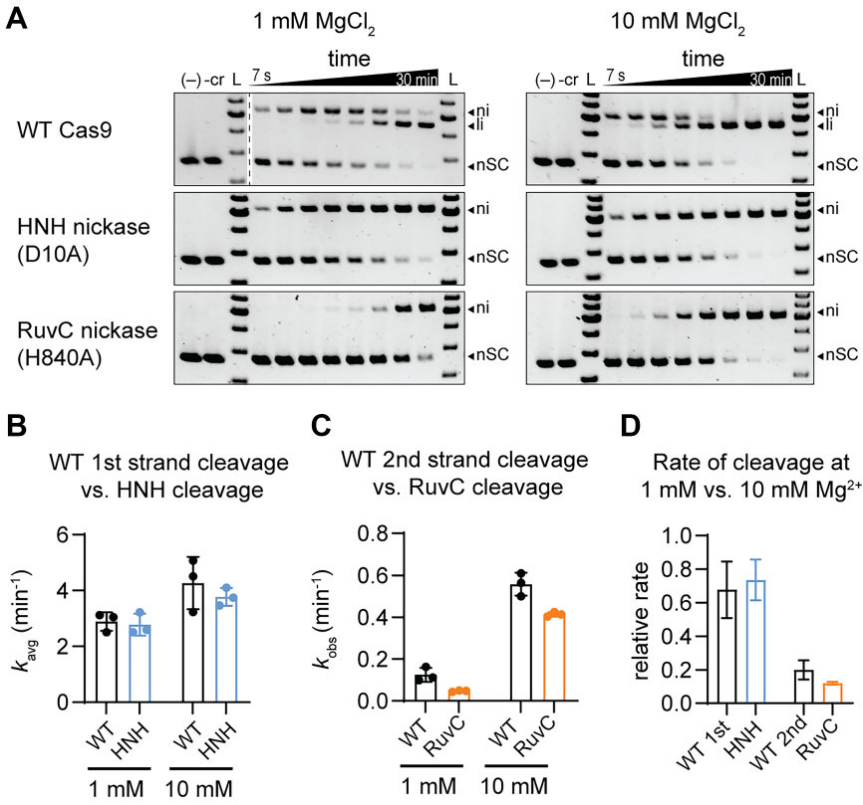
Cleavage by the Cas9 RuvC domain occurs more slowly than cleavage by the HNH domain at low Mg^2+^ concentration. (**A**) Agarose gels showing target plasmid cleavage time course for WT Cas9, HNH nickase, and RuvC nickase at 1 or 10 mM MgCl_2_ concentration. The two controls contain only plasmid (-) or Cas9 without guide RNA (-cr) incubated for the longest time point. Nicked (ni), linear (li), and negatively supercoiled DNA species are labeled. Time points: 7 s, 15 s, 30 s, 1 min, 2 min, 5 min, 15 min, and 30 min. (**B**) Rate constants for WT Cas9 first-strand cleavage (black) and cleavage by the HNH nickase (blue) at 1 or 10 mM MgCl_2_. Cleavage was biphasic and best fit to a double-exponential rate equation, as shown in Supplementary Fig. S1. The reported rate constants, *k*_avg_, were calculated as described in the “Materials and methods” section, and the underlying rate constants and amplitudes for the fast and slow phases are reported in Supplementary Fig. S1. Each dot represents the *k*_avg_ value from a single replicate (*n* = 3) and the error bars represent standard deviation. (**C**) Rate constants for WT Cas9 second-strand cleavage (black) and cleavage by the RuvC nickase (orange) at 1 or 10 mM MgCl_2_. The cleavage data was best fit to a single-exponential rate equation, as shown in Supplementary Fig. S1. The derived rate constant is reported as *k*_obs_. Each dot represents the *k*_obs_ value from a single replicate (*n* = 3) and the error bars represent standard deviation. (**D**) The relative rates for WT Cas9 cleaving the first or second strand and the HNH or RuvC nickase, colored as in panels (B) and (C). Relative rates were calculated by dividing the average *k*_avg_ or *k*_obs_ at 1 mM MgCl_2_ by the average rate constant at 10 mM MgCl_2_. The error bars represent standard deviations of the average values, with error propagation for division.

Cleavage of negatively supercoiled plasmid DNA by *Streptococcus pyogenes* (Sp) Cas9 has previously been observed to result in an initial nicked product, followed by formation of a linearized product [35, 42–44]. These two distinct products result from initial cleavage of the first DNA strand by one of the two Cas9 nuclease domains, followed by cleavage of the second DNA strand by the other domain. To determine which domain contributes to each cleavage event, we compared the rate of first- and second-strand cleavage by WT Cas9 to the rates of cleavage by two Cas9 nickases: an HNH nickase (D10A) that cleaves only the target strand and a RuvC nickase (H840A) that cleaves only the non-target strand [9] (Fig. 1A). First-strand cleavage by WT Cas9 and cleavage by the HNH nickase displayed biphasic kinetics, as has been previously reported for SpCas9 [18, 49]. We derived rate constants (*k*_avg_) that consider the contributions of both the fast and slow phases based on fitting the data to a double-exponential rate equation (Fig. 1B, Supplementary Fig. S1A, and see the “Materials and methods” section). In contrast, second-strand cleavage by WT Cas9 and cleavage by the RuvC nickase fit well to a single-exponential rate equation, yielding a single, observed rate constant (*k*_obs_) (Fig. 1C and Supplementary Fig. S1B).

We measured very similar rate constants at each Mg^2+^ concentration when comparing first-strand cleavage by WT Cas9 and cleavage by the HNH nickase (Fig. 1B). The rate constants for second-strand cleavage by WT Cas9 and cleavage by the RuvC nickase were also similar, although the RuvC nickase was slower, potentially due to variations in protein preparations or impairment of regulatory conformational changes due to the introduction of the H840A substitution (Fig. 1C). These results strongly suggest that first-strand cleavage by WT Cas9 corresponds to cleavage of the target strand by the HNH domain, while second-strand cleavage corresponds to cleavage of the non-target strand by the RuvC domain. Our results are consistent with sequential cleavage observed previously using shorter oligonucleotide DNA target substrates [18].

To determine the importance of Mg^2+^ for cleavage of each strand, we also compared rate constants determined at the two Mg^2+^ concentrations (Fig. 1D). The rate of first-strand cleavage by the HNH domain was decreased minimally at lower Mg^2+^ concentration, while the rate of second-strand cleavage by the RuvC domain was inhibited far more substantially. Collectively, these results suggest that second-strand cleavage by WT Cas9 is mediated by the RuvC domain and that this cleavage is highly sensitive to Mg^2+^ concentration.

### Nicking by the Cas9 HNH domain is sufficient to provide anti-phage defense for some targets

**Figure 2.**
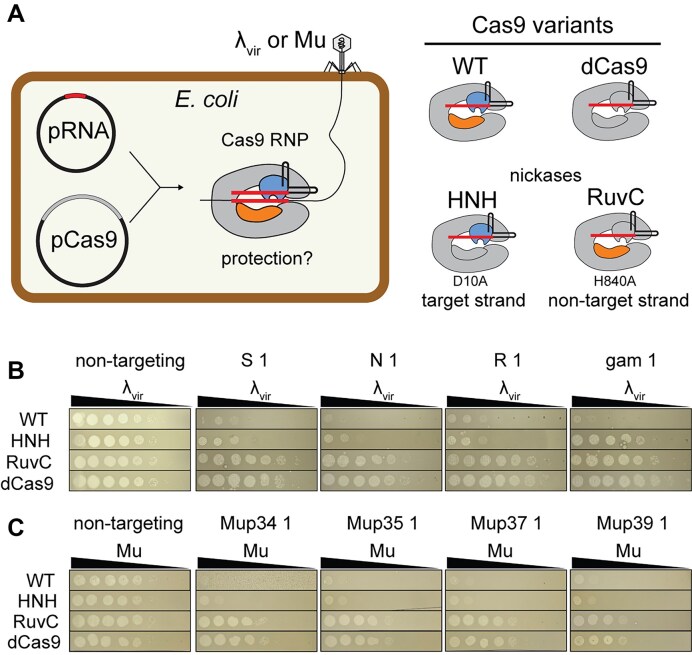
Nicking by the Cas9 HNH domain can provide resistance against phage. (**A**) Schematic of experimental design. Guide RNAs targeting various regions of the λ_vir_ or Mu phage genome were expressed from the pRNA plasmid and Cas9 variants shown on the right were expressed from pCas9. Phage spotting assays using (**B**) λ_vir_ or (**C**) Mu phage. A 10-fold dilution series of the indicated phage was spotted onto a lawn of *E. coli* BW25113 expressing WT Cas9, the HNH (D10A) nickase, the RuvC (H840A) nickase, or catalytically dead dCas9 and a guide RNA, indicated above the spotting assays. The non-targeting guide RNA did not match the phage genome, while the remaining four guides matched a region of the indicated protein-coding gene in the phage genome. Information on the guide RNA targets is provided in Supplementary Tables S1 and S2.

Our observation of RuvC metal-ion sensitivity led us to hypothesize that RuvC cleavage may occur slowly due to limited metal ion availability in cells. In this case, cleavage by the HNH domain could be sufficient to provide protection against phage under cellular conditions. To test this, we investigated whether either Cas9 nickase is sufficient to provide phage resistance. We introduced two plasmids for inducible expression of SpCas9 and an sgRNA in *E. coli* (Fig. 2A). We used an *E. coli* K12 strain (BW25113) that harbors a transcriptionally inactive type I-E CRISPR–Cas system that cannot provide significant protection against phage [50, 51]. These strains were infected with phage λ_vir_, a mutant of phage λ that is locked into the lytic cycle [45], or phage Mu, a temperate phage [46], using phage spotting assays (Fig. 2).

We compared the degree of anti-phage resistance provided by the two Cas9 nickases relative to WT Cas9 and catalytically dead Cas9 (dCas9, D10A/H840A), which can bind but not cleave targets [52–54]. We consistently observed lower plaquing efficiency for strains expressing WT Cas9 and a guide RNA targeting a gene within the λ_vir_ or Mu phage genome in comparison to a non-targeting control (Fig. 2B and C and Supplementary Tables S1 and S2). We observed a similar reduction in plaquing efficiency for strains expressing the HNH nickase and a targeting guide for most of the targets tested, with the exception of a guide RNA targeting the *gam* gene of λ_vir_. In contrast, we observed similarly high plaquing efficiencies for strains expressing the RuvC nickase or dCas9 using either non-targeting or targeting guide RNAs. Overall, these results suggest that the HNH nickase, but not the RuvC nickase, is capable of providing protection against multiple *E. coli* phages with either lytic or temperate life cycles.

### Comprehensive survey of protection by Cas9 variants against λ_vir_

Our initial experiments using phage spotting assays suggested that protection by the HNH nickase may vary for different targets tested against λ_vir_ (Fig. 2B). To further test this variability, we designed a total of 61 sgRNAs to target various essential and non-essential regions of the lambda phage genome (Supplementary Table S1). Targets were selected on both the template and coding strands, with several sgRNAs targeting the same or overlapping regions on either strand.

**Figure 3.**
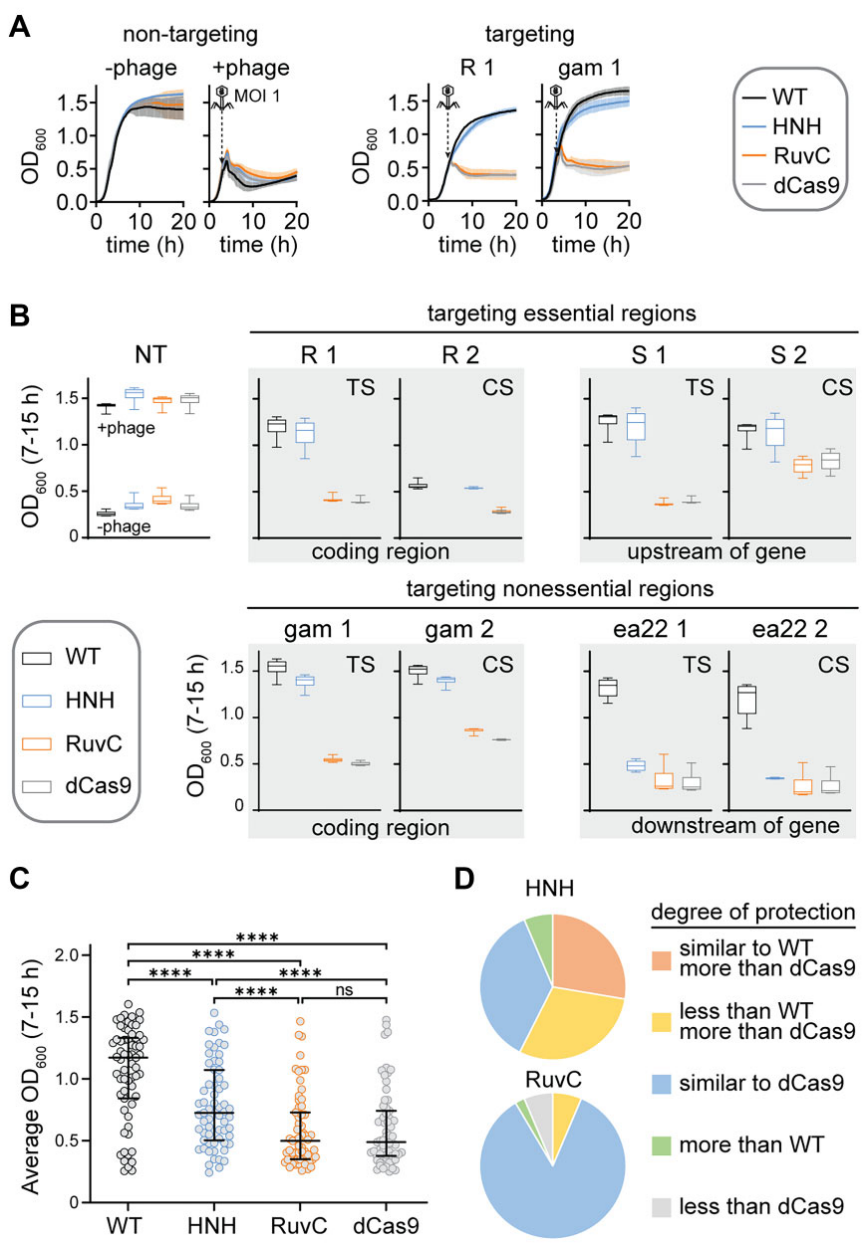
A large panel of guide RNAs reveals the extent of protection by the Cas9 HNH domain against λ_vir_. (**A**) Representative growth curves for *E. coli* BW25113 expressing pRNA and pCas9 without (−phage) or with (+phage) λ_vir_ infection at an MOI of 1. The growth curves on the left used a strain expressing a guide RNA that did not target the λ_vir_ genome. The growth curves on the right expressed guide RNAs targeting gene R or *gam* in the λ_vir_ genome. The average of three replicates is plotted with error bars representing standard deviation. Cell viability assays are shown in Supplementary Fig. S2. (**B**) Box plots of OD_600_ values over the 7–15 h time period of growth curves, summarizing protection by four sets of guide RNAs targeting the λ_vir_ genome. Error bars show the minimum and maximum values. Plots that are outlined together in gray represent guide RNAs that target the same or overlapping regions on either the template strand (TS) or coding strand (CS). Additional targets are shown in Supplementary Fig. S3. (**C**, **D**) Summary of protection by Cas9 variants for all tested sgRNAs. (C) The average OD_600_ value from 7 to 15 h for each of 61 targets was plotted for each Cas9 variant. Horizontal lines indicate the median and error bars represent the interquartile range (25th to 75th percentile). ns indicates no significant difference and **** indicates a *P*-value < .0001. (D) Pie charts indicating the degree of protection by the HNH nickase (top) or the RuvC nickase (bottom). Guide RNAs were assigned to each category by comparison of average OD_600_ values from 7 to 15 h. “Similar” indicates no statistical significance or a significant but small (<0.1) difference in the average OD_600_ values. “Less” or “more” indicates a statistically significant and large (>0.1) difference in the average OD_600_ values. Guide RNAs for which all four Cas9 variants provided similar protection were not included in the analysis (14 out of 61 guides). Categories for individual guides are listed in Supplementary Table S1.

We wished to test the panel of sgRNAs in a higher throughput manner using liquid cultures, which allowed the use of a 96-well plate. The growth of infected liquid cultures expressing Cas9 variants and sgRNA was monitored over time by measuring OD_600_ every 10 min (Fig. 3A). We confirmed that a decrease in OD_600_ corresponded to a loss of viable cells by measuring colony-forming units from samples collected prior to infection or 1 or 4 h post-infection (Supplementary Fig. S2). To easily compare protection by each variant, we plotted OD_600_ values at each time point from 7 to 15 h from three replicate cultures (Fig. 3B and Supplementary Fig. S3). During this time range, cultures in which Cas9 provides protection have a similar OD_600_ to uninfected cells, while cultures that have lysed have a substantially lower OD_600_ (Fig. 3B).

We observed strong protection by WT Cas9 for most targets (Fig. 3B and Supplementary Fig. S3). It has previously been reported that guide RNAs targeting the template strand of phage targets provide stronger protection than those targeting the coding strand [41, 55]. However, we did not observe any consistent trends in the relative amount of protection by WT Cas9 when targeting overlapping regions on the template versus the coding strand. We did not observe substantial protection by any Cas9 variants for a few targets (e.g. nu1, ea31, Supplementary Fig. S3), which may be due to misfolding of the sgRNA or high G/C content within the targeted region.

The amount of protection provided by dCas9 and the two Cas9 nickases varied significantly. For most targets, we observed no or low protection by dCas9, suggesting that transcriptional repression by dCas9 binding^38–41, 43^ is usually not sufficient to provide protection (Fig. 3B and Supplementary Fig. S3). For many targets, we observed more protection by the HNH nickase than the RuvC nickase. Comparison of the average OD_600_ from 7 to 15 h for all guides and all four Cas9 variants revealed that while the HNH nickase provided significantly less protection across all sgRNAs than WT Cas9, the HNH nickase also provided significantly more protection than either the RuvC nickase or dCas9 (Fig. 3C). Categorization of individual guides based on degree of protection relative to either WT or dCas9 revealed that the HNH nickase provided more protection than dCas9 for more than half of the guides, while there was no significant difference in protection between the RuvC nickase and dCas9 for the majority of guides (Fig. 3D). Overall, these results suggest that nicking by the Cas9 HNH domain, but not the RuvC domain, can be sufficient to provide protection for some, but not all, targets across a phage genome.

### Genomic deletions allow phage escape from WT Cas9 but not nickases

**Figure 4.**
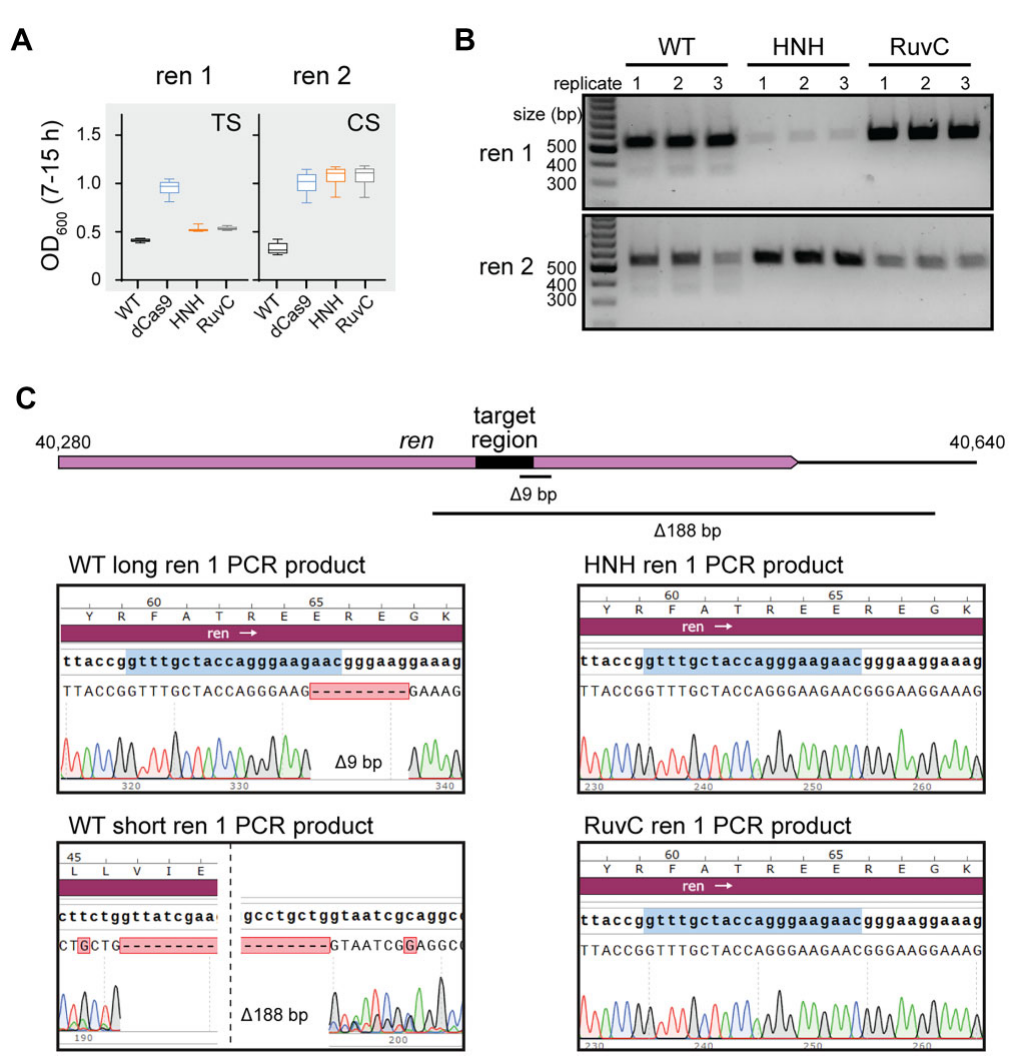
Nicking can provide protection while preventing deletion of target region. (**A**) Box plots (as in Fig. 3B) for *E. coli* cells expressing Cas9 variants and one of two guide RNAs targeting the same region on the template (TS) or coding (CS) strand of the *ren* gene, a non-essential gene in the λ genome. (**B**) Agarose gel for PCR amplicons of the *ren* gene from phage recovered following growth of cultures plotted in panel (A). (**C**) Sanger sequencing chromatograms for *ren* PCR amplicons for cultures expressing WT Cas9, HNH nickase, or RuvC nickase. PCR amplicons of ∼550 bp were sequenced for the long WT Cas9 PCR product and both nickase products. The short WT Cas9 PCR was purified from the band at ∼350 bp on the gel in panel (B).

Surprisingly, for three targets, we observed better protection by the HNH nickase than WT Cas9. Two of these sgRNAs, targeting overlapping regions of the non-essential *ren* gene on either the template or coding strand, provided no protection when paired with WT Cas9 and partial protection by the HNH nickase or by all three other Cas9 variants (Fig. 4A). It has previously been observed that Cas effector cleavage can result in mutagenesis within the target region of phage genomes, including point or deletion mutations [36, 39–41]. Such mutations could allow phages to escape Cas9-mediated immunity, resulting in the lack of protection by WT Cas9.

To determine whether Cas9 cleavage resulted in target mutations, we PCR-amplified phage genomic regions containing the *ren* targets following challenge with WT Cas9 or either Cas9 nickase. We observed a single product of the expected size for phages challenged with the nickases, but an additional light smear of products smaller than the expected size when WT Cas9 was used (Fig. 4B). Sequencing of the PCR amplicons revealed that phages challenged with WT Cas9 contained a mixture of different deletions in the targeted region. The full-length amplicon contained a short 9-bp deletion that removed the PAM and first three nucleotides of the seed. The short PCR amplicon contained a larger 188-bp deletion that removed the entire target site. In contrast, both Cas9 nickases retained the original target sequence. Overall, these results suggest that nicking by Cas9 may allow enhanced protection by reducing the tendency of phages to develop escape mutations following Cas9 targeting.

### Target strand nicking reduces phage DNA abundance

**Figure 5.**
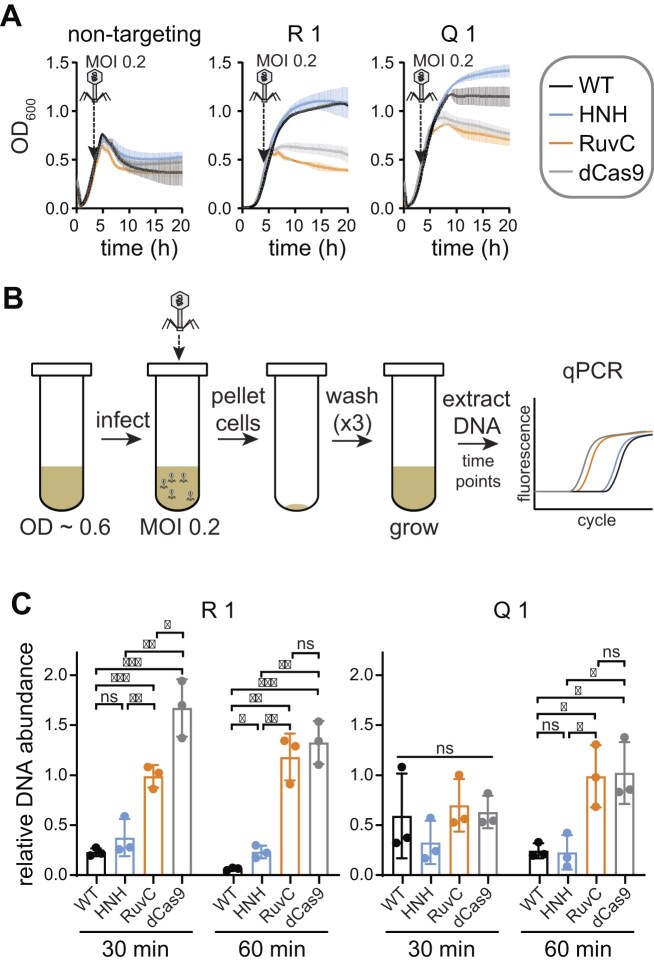
HNH domain nicking reduces phage DNA abundance. (**A**) Growth curves for cultures used for qPCR analysis. *Escherichia coli* BW25113 expressing a Cas9 variant and a non-targeting control guide RNA, or guide RNAs targeting gene R or Q in the λ genome, were infected with λ_vir_ at an MOI of 0.2. (**B**) Schematic of phage infection to ensure a single infection cycle. *Escherichia coli* BW25113 expressing Cas9 variants and guides used in panel (A) were grown to mid-log phase. Phage was added at an MOI of 0.2 and incubated for 10 min to allow adsorption, then cells were pelleted and washed with fresh LB three times. Cells were grown for 60 min, and phage DNA was assayed using qPCR at 30 and 60 min. (**C**) Relative abundance of λ_vir_ DNA in cultures expressing each Cas9 variant. Relative DNA abundance was calculated by normalizing Ct values for cultures expressing a targeting guide RNA versus cultures expressing the non-targeting guide (see the “Materials and methods” section). The average value of three replicates is shown, with individual data points shown as dots. Error bars represent standard deviation. *P*-values were determined using an unpaired, two-tailed *t*-test. ns: *P*> .05, *: *P* < .05, **: *P* < .01, ***: *P* < .001.

We next asked whether target strand nicking by the HNH nickase results in a loss of accumulation of phage DNA following infection. Nicking has previously been shown to inhibit phage genome replication [56] and could potentially lead to degradation of phage DNA. To test whether nicking by Cas9 nickases reduces phage DNA accumulation, we used qPCR to measure phage DNA abundance upon challenge by each Cas9 variant. We selected two guide RNAs (R1 and Q1) that conferred protection only when paired with WT Cas9 or the HNH nickase (Fig. 5A). To measure phage DNA abundance, mid-log cultures were infected with λ_vir_ at an MOI of 0.2 for 10 min (Fig. 5B). The cells were then pelleted, washed three times, and grown in fresh media. An aliquot of the cultures was harvested immediately after adsorption and following 30 or 60 min of growth. Cells infected at an MOI of 0.2 did not lyse within 60 min (Fig. 5A), indicating that the selected time points occur within the timeframe of a single infection cycle. The harvested samples were boiled to prevent further DNA replication, then were used as DNA templates for qPCR (see the “Materials and methods” section). Both the phage and host chromosomal DNA were analyzed by qPCR to account for any differences in host replication that may occur upon phage infection. No differences in host DNA Ct values were observed over the three time points (Supplementary Table S6).

Relative to cultures expressing a non-targeting guide, phage DNA decreased significantly in cells expressing WT Cas9 and either the R1 or Q1 guide (Fig. 5C). We observed similarly low relative amounts of phage DNA from cultures expressing the HNH nickase and either guide. Phage DNA was significantly higher for the dCas9 and RuvC cultures at both time points for the R1 guide, but only at the 60 min time point for the Q1 guide, suggesting that Cas9 binding may have initially inhibited phage replication in cultures expressing the Q1 guide. Consistently, we observed a longer period of growth following addition of phage to liquid cultures expressing dCas9 and the RuvC nickase bearing the Q1 guide than for cultures with a non-targeting guide (Fig. 3A). Overall, these results suggest that nicking by the HNH domain is sufficient to inhibit phage DNA accumulation, but that Cas9 binding or nicking by the RuvC domain does not substantially impair phage replication over the course of an infection cycle.

### Non-target strand nicking is necessary for Cas9 turnover by RNA polymerase

Our phage assays suggest that nicking of the target strand by the HNH domain can be sufficient to provide protection against phage on par with WT Cas9 for a subset of targets, but that nicking of the non-target strand by the RuvC domain can only improve protection and is not sufficient to provide protection in the absence of a target strand nick. One potential explanation for the difference between target and non-target strand nicking is the ability for Cas9 to turn over following target cleavage. Although Cas9 typically remains bound to its product following cleavage, it has previously been demonstrated that RNA polymerase can dislodge Cas9 from the template strand of a DNA substrate [55]. Conversion of Cas9 into a multi-turnover enzyme has been suggested to improve protection against phage. Alternatively, rapid turnover of Cas9 following first-strand cleavage by a single nuclease domain may prevent formation of a DSB in the event that second-strand cleavage occurs in a delayed manner. Thus, it is possible that differences between turnover of HNH and RuvC nickases may account for the differences in protection observed for these Cas9 variants.

**Figure 6.**
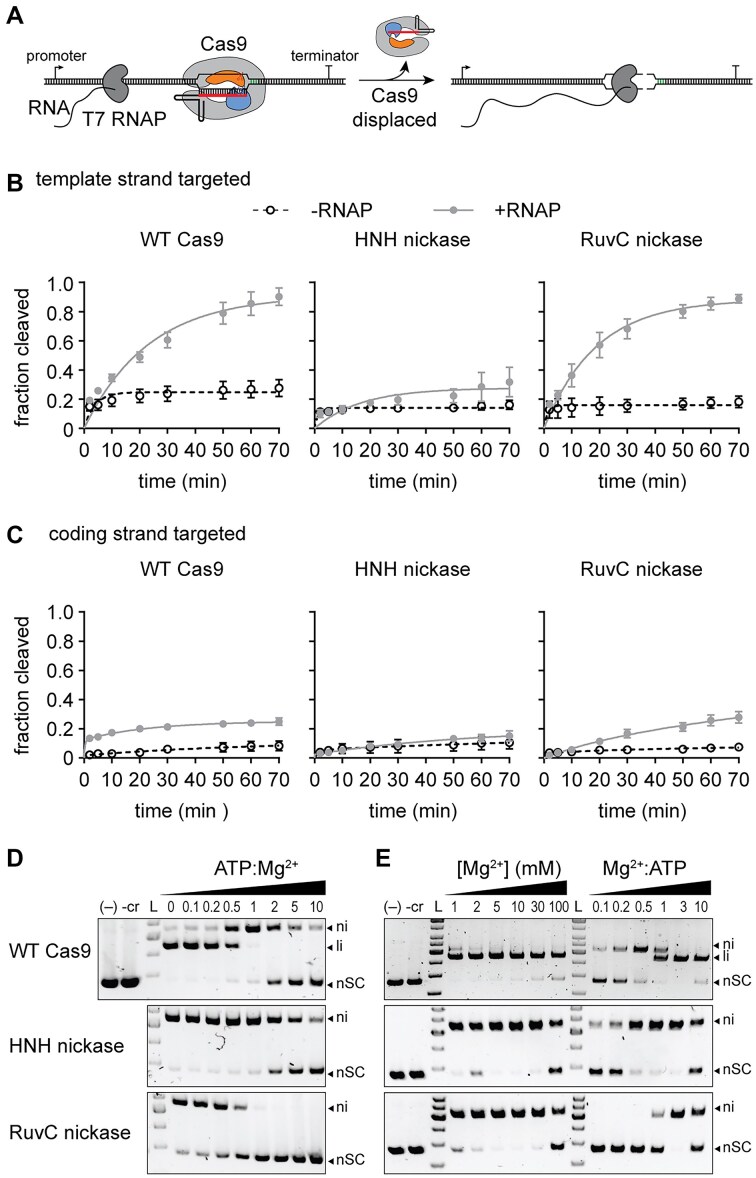
Target strand nicking prevents Cas9 turnover and occurs under physiological metal ion conditions. (**A**) Schematic of Cas9 turnover assay. The Cas9 target was located on the template strand downstream of a T7 RNAP promoter. WT Cas9 typically remains bound to the target following cleavage, but can be dislodged by RNA polymerase transcription of the target region. Fraction cleaved over time for the (**B**) template or (**C**) coding strand by WT Cas9, HNH nickase, or RuvC nickase in the absence (open circles, dashed lines) or presence (closed gray circles, solid line) of RNA polymerase. The average of three replicates is shown and error bars represent standard deviation. Representative gels are shown in Supplementary Fig. S2. (**D**) Target plasmid cleavage at varying ATP:Mg^2+^ ratios. The MgCl_2_ concentration was 1 mM and each reaction was incubated for 30 min. (**E**) Target plasmid cleavage at varying MgCl_2_ concentrations in the absence or presence of 10 mM ATP. Each reaction was incubated for 30 min. The loss of cleavage at 100 mM MgCl_2_ may be due to destabilization of the Cas9 structure or effects on nucleic acid structure.

To test this, we performed Cas9 cleavage assays in the absence and presence of T7 RNAP (Fig. 6A). The substrate plasmid contained a T7 promoter upstream of the target site, which was located either on the template or the coding strand. We performed cleavage assays using a slight excess of the plasmid over Cas9-guide RNA complex. In the absence of T7 RNAP, all three Cas9 variants cleaved 5–20 percent of the target DNA, consistent with an inability of the enzymes to turn over following a single round of cleavage (Fig. 6B and C and Supplementary Fig. S4). The fraction cleaved by WT Cas9 increased substantially upon addition of T7 RNAP when targeting the template strand, indicating that Cas9 is dislodged by T7 RNAP, allowing for further cleavage of the target (Fig. 6B and Supplementary Fig. S4A). We observed a similar turnover rate for the RuvC, but not the HNH nickase, suggesting that nicking of the non-target strand is necessary to allow Cas9 turnover by RNAP. We similarly observed more turnover for WT Cas9 and the RuvC nickase when targeting the coding strand, albeit to a much smaller degree than for the template strand (Fig. 6C and Supplementary Fig. S4B). It has previously been observed that RuvC cleavage exposes the 3′ end of the non-target strand, while the target strand remains hybridized with the crRNA after cleavage by the HNH domain [57]. Together with our turnover results, these observations suggest that the exposed nicked non-target strand is necessary for RNA polymerase to dislodge Cas9 from target DNA following cleavage by the RuvC domain, similar to a model proposed for Cas9 turnover by the human replication fork remodeler HLTF published while our manuscript was in preparation [58]. Overall, these results suggest that following an initial nick by the HNH domain, Cas9 can remain bound to the target, allowing delayed cleavage by the RuvC domain due to its slower cleavage kinetics when Mg^2+^ is limiting.

### Competition for metal ion binding significantly impairs cleavage by the RuvC domain

Our combined *in vitro* and anti-phage protection results suggest that the Cas9 RuvC domain may act as a safeguard to ensure full protection following an initial nick by the HNH domain. The rate of cleavage by RuvC in cells is likely to depend on the availability of free Mg^2+^. Cellular Mg^2+^ concentrations are estimated in the 10–100 mM range, but much of this is expected to be bound by various biomolecules in cells [59, 60]. For example, nucleotide triphosphates (NTPs) can chelate metal ions with a *K*_d_ in the sub-mM range [61]. Thus, enzymes like Cas9 that are highly sensitive to metal ion concentrations may also be sensitive to concentrations of other metal-binding biomolecules in cells. To simulate this, we performed plasmid cleavage assays in the presence of 1 mM Mg^2+^ and increasing concentrations of ATP (Fig. 6D). We observed a substantial loss of cleavage by the RuvC domain at sub-stoichiometric amounts of ATP:Mg^2+^, and a complete loss of cleavage at equimolar amounts of ATP and Mg^2+^. In contrast, the HNH domain was able to cleave the DNA until the ATP concentration exceeded Mg^2+^. We observed similar results when we titrated Mg^2+^ in the absence or presence of a constant concentration of ATP (Fig. 6E). Together with experiments in Fig. 1, our results suggest that the RuvC domain of Cas9 is highly sensitive to metal ion conditions as well as to the concentration of other biomolecules, which may result in decreased rates of RuvC cleavage when metal ion availability is low in cells.

## Discussion

**Figure 7.**
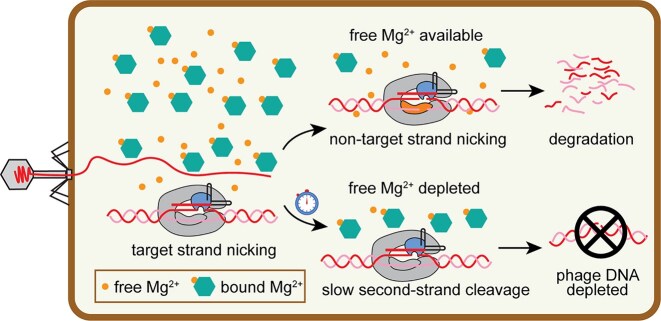
Slow second-strand cleavage during Cas9-mediated phage defense. Model for the effect of RuvC metal-ion sensitivity on Cas9-mediated phage defense. Under typical cellular conditions, cleavage of the target strand by the HNH domain occurs more rapidly than cleavage of the non-target strand by the RuvC domain. When free Mg^2+^ is highly available, non-target strand nicking can occur shortly after target strand nicking, inducing a DSB and promoting target degradation. Under conditions of low metal-ion availability, second-strand cleavage is delayed. Nicking by the HNH domain may be sufficient to inhibit phage DNA accumulation by blocking replication or promoting degradation, allowing protection against phage in the absence of DSB formation for some targets.

DNA-targeting Cas effectors can provide robust defense against phage by directly damaging viral DNA. Canonically, Cas9 and Cas12 effectors are thought to induce double-strand breaks that enable degradation of the invading genome through the activity of other cellular nucleases [9–11]. However, both Cas9 and Cas12a can be prone to nicking of DNA [31–32, 34–35, 42–44], raising the question of whether Cas effectors can provide protection against phage when inducing only a DNA nick. We found that *in vitro* cleavage by the Cas9 HNH domain occurs more rapidly than cleavage by the RuvC domain at low, physiological metal ion concentrations, especially in the presence of other biomolecules that can compete for metal ion binding. Consistently, in phage infection assays, we observed that for some targets, nicking by the HNH domain, but not the RuvC domain, can be sufficient to provide protection against phage. Together, these results suggest that Cas9 may initiate anti-phage defense through rapid cleavage of the target strand by the HNH domain, especially under conditions where free Mg^2+^ is limited (Fig. 7). The initial nick by the HNH domain can be sufficient to prevent phage DNA accumulation depending on the type of infecting phage or genomic target. However, second-strand cleavage by the RuvC domain was necessary for full protection against λ_vir_ for the majority of targets, indicating that formation of a DSB is often required for full protection. We observed that Cas9 is not readily dislodged from the target DNA following cleavage by the HNH domain. This suggests that Cas9 may remain bound to the target following first-strand cleavage, allowing for second-strand cleavage to occur in a delayed manner, to ensure complete protection (Fig. 7).

It is important to note that our study was performed under heterologous conditions, and it is possible that Cas9 expression level or regulation by other host factors may impact the degree of protection afforded by either Cas9 nickase. Nevertheless, slow RuvC cleavage is an intrinsic property of SpCas9 and is likely to occur in any environment in which Mg^2+^ concentration is limiting. The differential effects of metal ion concentration on the HNH and RuvC domains may be due to the metal dependence of conformational changes or R-loop propagation, both of which have been implicated in activating cleavage by the RuvC domain [18, 26–27]. In addition, the different cofactor requirements of the two domains may account for their variable sensitivity to metal-ion availability. The HNH domain is thought to require only a single metal ion, while the RuvC domain requires two metal ions [18, 62–63]. It is possible that RuvC may require a third, more transiently bound metal ion, as has been detected for other enzymes in the RNase H-like superfamily [64, 65]. This requirement could account for the extreme sensitivity to competition for metal ion binding we observed in the presence of competing biomolecules.

Slow second-strand cleavage may be beneficial during phage infection because it can reduce the rate of phage DNA accumulation while decreasing the likelihood of escape mutations arising following DSB formation at the target site. Indeed, we observed that nicking by the HNH domain reduced the amount of phage DNA present during an infection cycle to a similar degree as WT Cas9, while also preventing deletion mutations that otherwise arose upon targeting of non-essential genomic regions by WT Cas9. We only observed escape from WT Cas9 for a small number of sgRNAs, potentially because of the overexpression conditions used in our study. Future studies could explore the relative ability of phages to escape WT and Cas9 nickases under conditions more amenable to phage escape.

It has previously been observed that Cas9 provides better protection when targeting the template strand than the coding strand, potentially due to the turnover of Cas9 by RNA polymerase [40, 55]. However, when testing a larger pool of guide RNAs targeting overlapping regions on the template and coding strand, we did not observe a consistent strand bias in Cas9-mediated immunity. Furthermore, the HNH nickase often exhibited efficient phage resistance, although we did not observe turnover of the HNH nickase by RNA polymerase. In contrast, the RuvC nickase, although dislodged by RNA polymerase, did not provide protection in our phage assays. These results suggest that Cas9 turnover is not essential for phage resistance. It is also possible that the removal of the RuvC nickase allowed rapid repair of the nicked DNA, compromising the effectiveness of the RuvC nickase in combating phage infections. Similarly, it has been proposed that turnover of the H840A RuvC nickase by the replication fork remodeler HLTF allows rapid repair of nicks, preventing effective genome editing by the RuvC nickase in human cells [58].

Other Cas endonucleases, including Cas12a, have also been observed to nick DNA in a metal-dependent manner [24, 36]. We previously observed that mutations arise preferentially in the PAM-distal region upon targeting by Cas12a [36]. PAM-distal mutations cause substantial target-strand cleavage defects at lower Mg^2+^, which are alleviated at higher metal ion concentrations [24]. While it remains unclear whether nicking by Cas12a is sufficient to provide protection against phage, it is notable that Cas12a nicks the non-target rather than the target strand. Thus, it is possible that Cas12a is more susceptible to escape via PAM-distal mutations than Cas9, as we have previously observed [36].

## Supplementary Material

gkaf900_Supplemental_Files

## Data Availability

All data generated during this study are included in this published article and its supplementary information files. The Ct values for the qPCR data are provided in Supplementary Fig. S6.

## Notes

Present address: Wadsworth Center, New York State Department of Health, Albany, NY, United States

Present address: Department of Hematology, St. Jude Children’s Research Hospital, Memphis, TN, United States
