# Supplementary material for: Rapid CRISPR–Cas9 target-strand nicking can provide phage resistance by reducing DNA abundance
Source: Nucleic Acids Res. 2025 Sep 23;53(18):gkaf900. doi: 10.1093/nar/gkaf900 (PMC12455587; doi:10.1093/nar/gkaf900)
Supplement: gkaf900_Supplemental_Files [file gkaf900_supplemental_files.zip › Cas9 nicking-manuscript-SI.pdf]

A

$$y = A_{fast} \times (1 - e^{(-k_{fast} \times x)}) + A_{slow} \times (1 - e^{(-k_{slow} \times x)})$$

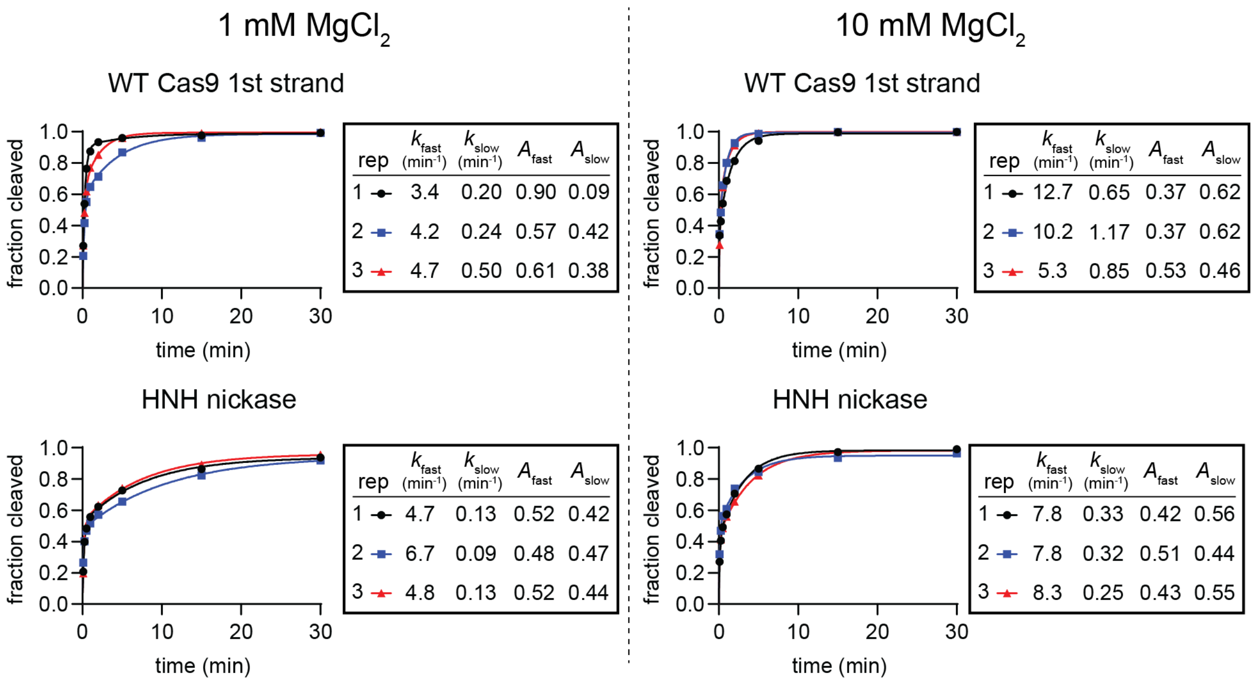

B

$$y = A \times (1 - e^{(-k_{obs} \times x)})$$

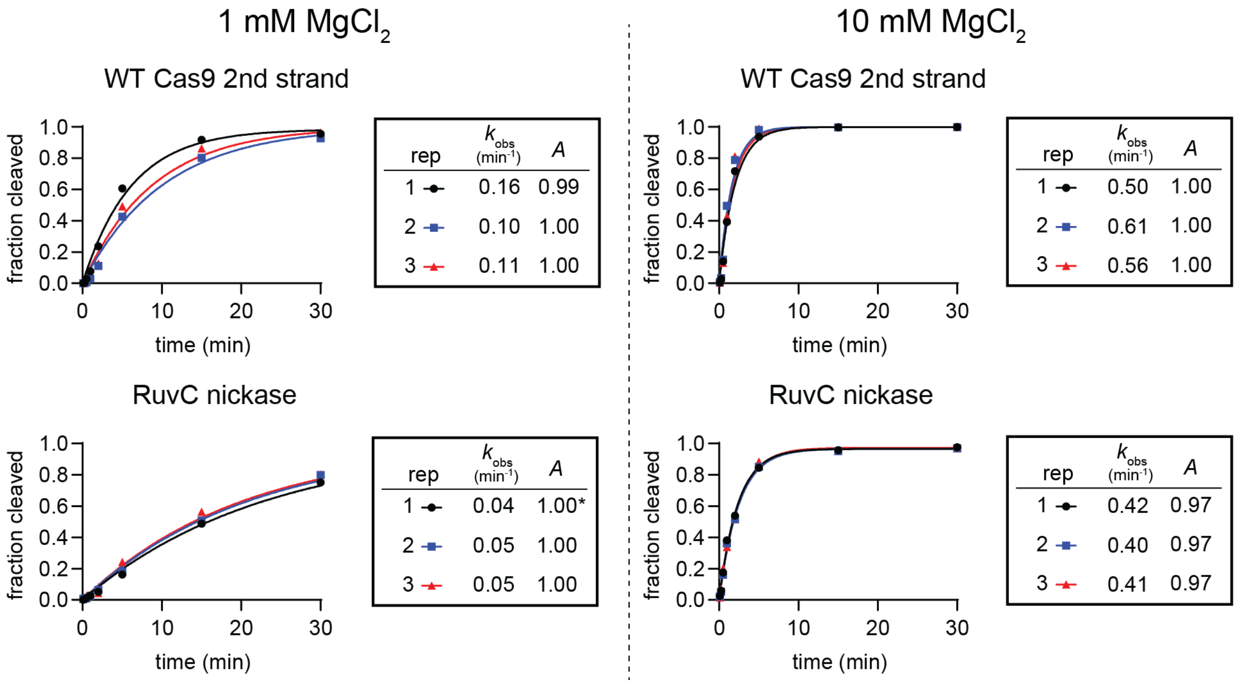

Figure S1: Quantification and fitting of Cas9 cleavage assays.

A) Plots of fraction cleaved versus time for the first cleavage event by Cas9 or nicking by the HNH nickase at 1 or 10 mM $MgCl_2$. The data fit best to a double-exponential rate equation, shown at the top of the panel. The values for rate constants and amplitudes for the fast (k_{fast} and A_{fast}, respectively) and slow (k_{slow} and A_{slow}, respectively) phases are indicated for three replicates to the right of each graph. These values were used to determine k_{avg} reported in Fig. 1 as described in the Methods.

B) Plots of fraction cleaved versus time for the second cleavage event by Cas9 or nicking by the RuvC nickase at 1 or 10 mM $MgCl_2$. The data were fit to a single-exponential rate equation, shown at the top of the panel. The rate constant (k_{obs}) and amplitude (A) are indicated for three replicates to the right of each graph.

For all graphs, the initial y value was constrained to 0, and the amplitude was constrained to less than 1. An asterisk indicates that the value hit the constraint, which occurred for the amplitude of the first replicate for the RuvC nickase at 1 mM $MgCl_2$.

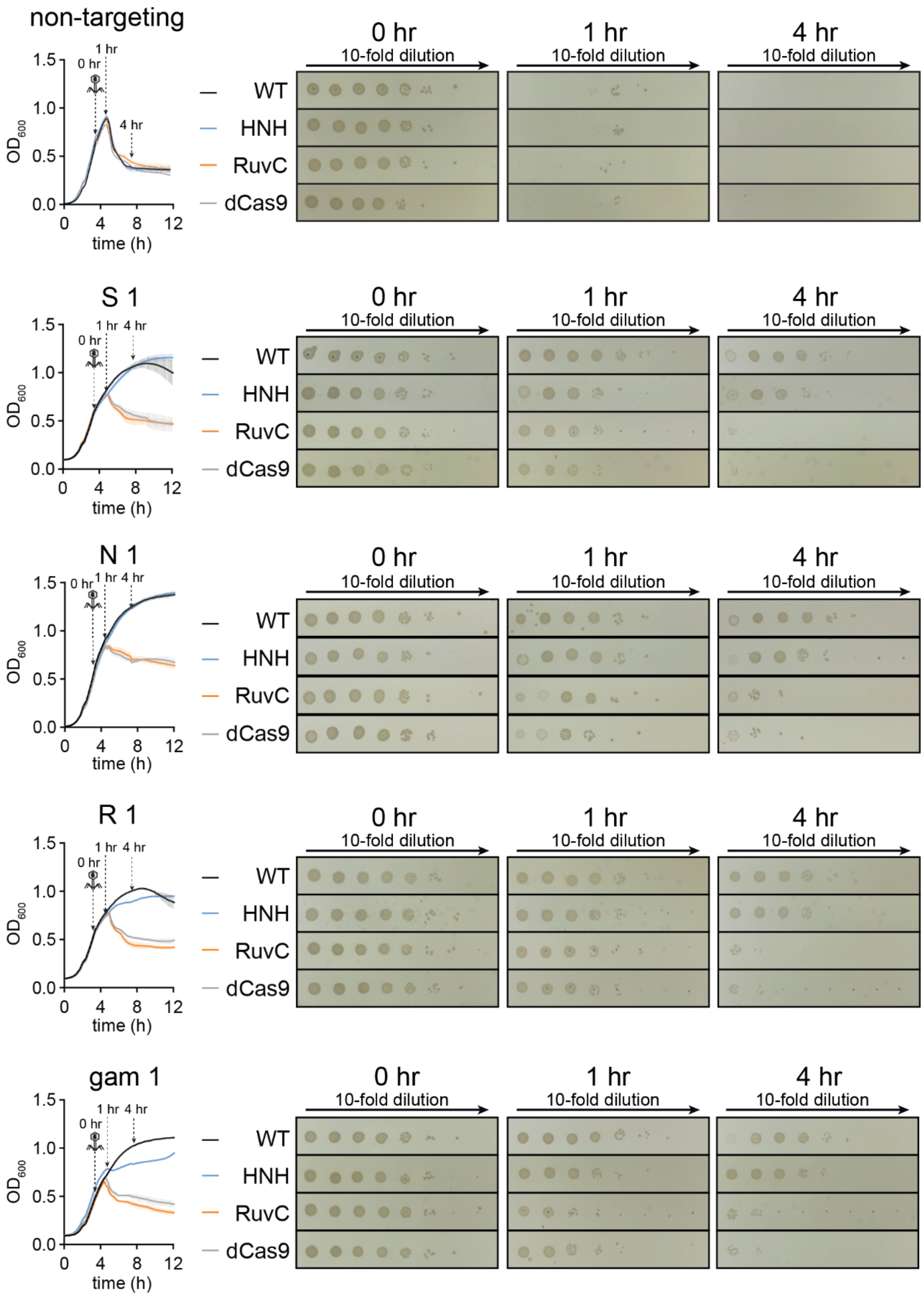

Figure S2: Cell viability assays for liquid cultures of *E. coli* infected with λ_{vir}. Growth curves plotting OD600 versus time are shown on the left for *E. coli* cultures expressing WT Cas9

(black), the HNH nickase (blue), the RuvC nickase,(orange) or dCas9 (gray). Each culture also expressed a guide RNA indicated above the graph. Phage was added to the culture at the timepoint labeled “0 hr”. Cells were harvested from cultures at each of the timepoints indicated on the growth curves. After washing the cells, a 10-fold dilution series of the cells were spotted onto LB plates with appropriate antibiotic selection, shown on the right for the three time points for each Cas9 variant.

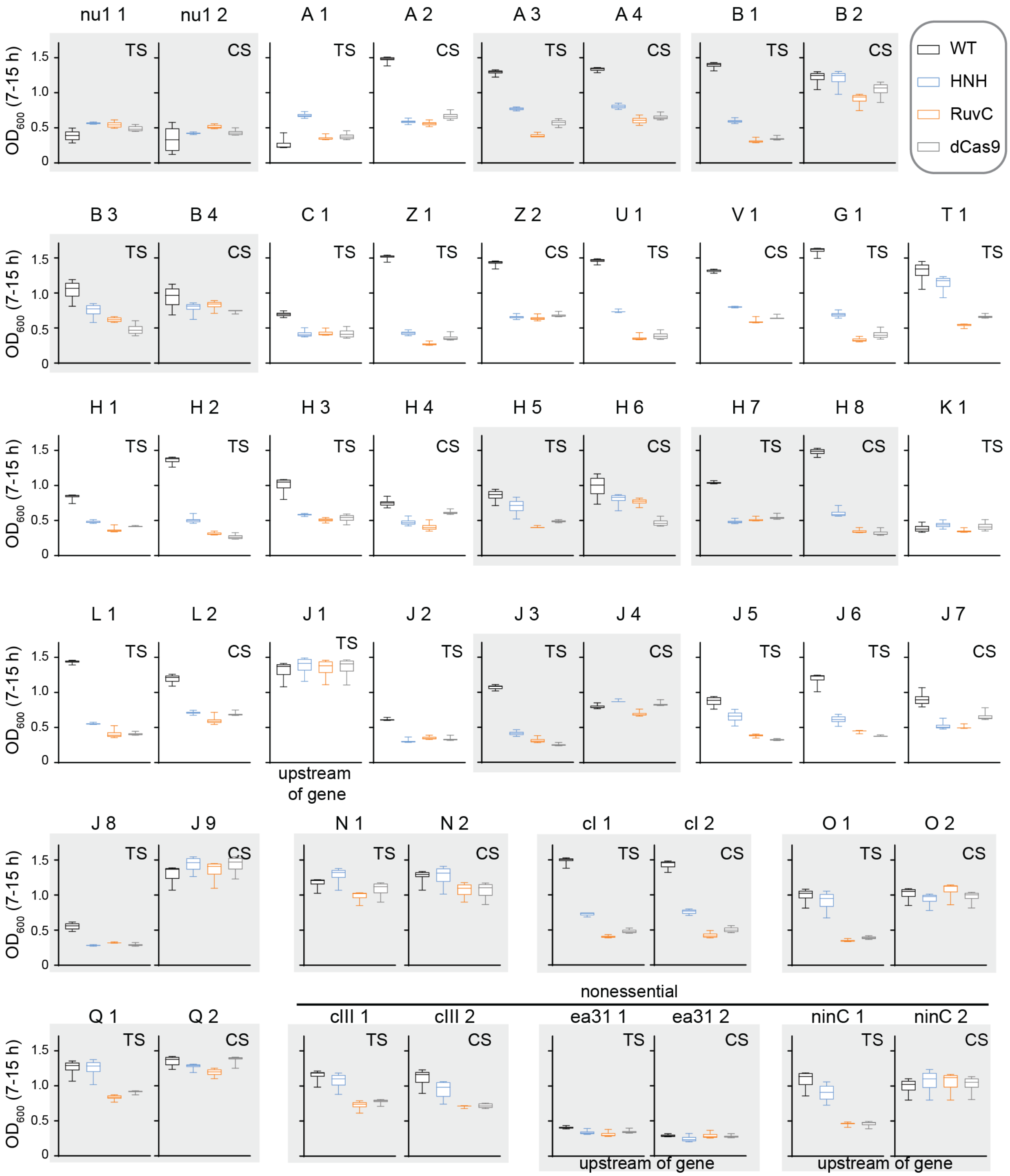

Figure S3: Protection by Cas9 variants for additional guide RNAs.

Box plots of OD600 values over the 7-15 h time period of growth curves, summarizing protection by four Cas9 variants using different guide RNAs against λ_{vir}. The gene targeted by the guide RNA is indicated. Plots that are outlined together in gray represent guide RNAs that

target the same or overlapping regions on either the template strand (TS) or coding strand (CS). Guide RNAs targeting non-essential genes or non-coding regions are indicated. All gene targets are provided in Table S1.

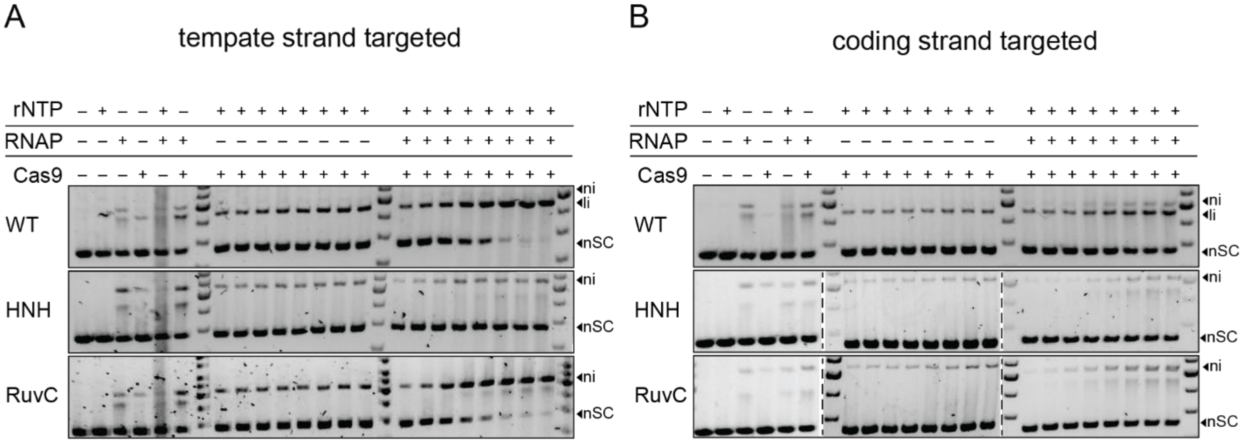

Figure S4: Gels showing Cas9 turnover by T7 RNA polymerase

Cleavage assays were performed with a slight excess of plasmid DNA over Cas9. In the absence of RNAP, only a fraction of the DNA is cleaved due to the lack of Cas9 turnover. When RNAP is added in the presence of rNTPs, Cas9 can turnover under some conditions tested, resulting in full cleavage of the DNA at longer time points.

A. The template strand of the target plasmid was targeted.

B. The coding strand of the target plasmid was targeted.

Supplementary Tables

Table S1: List of λ genome targets.

Table S2: List of Mu genome targets.

Table S3: Primers for *cas9* mutagenesis and plasmids for Cas9 expression.

Table S4: Oligonucleotides for tracrRNA and crRNA synthesis and for cloning plasmid targets for cleavage assays.

Table S5: Primers used for qPCR.

Table S6: Ct values from qPCR assays.
